# Proteomic profiling of plasma-derived small extracellular vesicles: a novel tool for understanding the systemic effects of tick burden in cattle

**DOI:** 10.1093/jas/skac015

**Published:** 2022-01-19

**Authors:** Natalie Turner, Pevindu Abeysinghe, Hassendrini Peiris, Kanchan Vaswani, Pawel Sadowski, Nick Cameron, Nathanael McGhee, Jayden Logan, Murray D Mitchell

**Affiliations:** 1 Centre for Children’s Health Research (CCHR), Queensland University of Technology (QUT), South Brisbane, QLD 4101, Australia; 2 School of Biomedical Sciences, Central Analytical Research Facility (CARF), Brisbane City, QLD 4000, Australia; 3 Nindooinbah, Beaudesert, QLD 4285, Australia

**Keywords:** cattle, exosomes, extracellular vesicles, mass-spectrometry, tick resistance

## Abstract

Cattle ticks pose a significant threat to the health and profitability of cattle herds globally. The investigation of factors leading to natural tick resistance in cattle is directed toward targeted breeding strategies that may combat cattle tick infestation on the genetic level. Exosomes (**EX**s), small extracellular vesicles (**EV**s) of 50 to 150 nm diameter, are released from all cell types into biofluids such as blood plasma and milk, have been successfully used in diagnostic and prognostic studies in humans, and can provide essential information regarding the overall health state of animals. Mass spectrometry (**MS**) is a highly sensitive proteomics application that can be used to identify proteins in a complex mixture and is particularly useful for biomarker development. In this proof of principle study, EXs were isolated from the blood plasma of cattle (*Bos taurus*) with high (**HTR**) and low tick resistance (**LTR**) (*n* = 3/group). Cattle were classified as HTR or LTR using a tick scoring system, and EXs isolated from the cattle blood plasma using an established protocol. EXs were subjected to MS analysis in data-dependent acquisition mode and protein search performed using Protein Pilot against the *B. taurus* proteome. A total of 490 unique proteins were identified across all samples. Of these, proteins present in all replicates from each group were selected for further analysis (HTR = 121; LTR = 130). Gene ontology analysis was performed using PANTHER GO online software tool. Proteins unique to HTR and LTR cattle were divided by protein class, of which 50% were associated with immunity/defense in the HTR group, whereas this protein class was not detected in EXs from LTR cattle. Similarly, unique proteins in HTR cattle were associated with B-cell activation, immunoglobins, immune response, and cellular iron ion homeostasis. In LTR cattle, unique exosomal proteins were associated with actin filament binding, purine nucleotide binding, plasma membrane protein complex, and carbohydrate derivative binding. This is the first study to demonstrate that MS analysis of EXs derived from the blood plasma of HTR and LTR cattle can be successfully applied to profile the systemic effects of tick burden.

## Lay Summary

Cattle ticks are a significant burden to cattle industries globally. Current methods to treat cattle ticks are costly and inefficient in the long term. It has been noted that while some cattle may exhibit a natural resistance to ticks, others carry a heavy tick burden. The study of small extracellular vesicles, or exosomes (EXs), isolated from cattle blood plasma provides a noninvasive way of analyzing changes at the cellular level and may be of use in understanding the systemic effects of tick burden or factors leading to natural resistance. The aim of this study was to assess high (HTR) and low tick resistance (LTR) cattle identified using a tick burden scoring system by analyzing the protein content of circulating EXs via qualitative proteomics analysis. We found that a class of proteins related to defense/immunity comprised 50% of proteins unique to HTR cattle, while this protein class was not detected in proteins unique to LTR cattle. Additionally, epidermal growth factor–calcium-binding protein domains were 2-fold increased in LTR cattle compared with HTR cattle, indicating a possible mechanism for widespread metabolic change. This is the first study to employ proteomic analysis of exosomal cargo as an approach to understanding the systemic effects of tick burden in cattle.

## Teaser

Small extracellular vesicles (EVs) of size ~50 to 150 nm have been isolated and enriched from the blood plasma of cattle exhibiting high and low natural resistance to tick infestation. Proteomic analysis of these small EVs by mass spectrometry has provided valuable information regarding the systemic effects, and particularly the immune challenges, of tick burden on animals exhibiting low tick resistance.

## Background

Cattle ticks and tick-borne diseases represent a huge burden to cattle industries globally, with 80% of the world’s cattle situated in areas of tick prevalence (de Castro, 1997). In Australia, the financial losses to industry associated with cattle ticks are estimated to exceed $160 million annually (Mahony, 2021). While there is evidence to suggest that susceptibility to tick infestation is cattle species dependent, the mechanisms underlying natural resistance to ticks remain unclear (Robbertse et al., 2017). There is some evidence to suggest that the ability of the host to acquire immunity to tick infestation over time encompasses both adaptive and innate immune response; however, it is clear that this immunity fails to develop in a significant portion of cattle (Rechav et al., 1991; Jonsson et al., 2014). Strategies to combat cattle tick infestation include acaricides and vaccine-based treatments; however, these are not long-term solutions due to sustainability and environmental concerns, and promotion of tick resistance to acaricide treatment (de Castro, 1997; Jonsson, 2006). 

Studies investigating natural resistance in cattle to tick infestation include the sampling of blood and skin from cattle to perform genetic and immunological studies with the hope of developing biomarkers of natural resistance (Bagnall et al., 2009; Akbar et al., 2015; Marima et al., 2020). Genetic, proteomic, and peptidomic studies of cattle ticks have also been employed to gain an understanding of the parasite–host relationship that underlies tick infestation (Garcia et al., 2020; Xiong et al., 2020; Sajiki et al., 2021). The sampling of blood plasma is ideal as a diagnostic or prognostic tool as it is relatively simple to obtain and carries information at the systemic level. Secreted cell proteins and genetic material are released into the bloodstream, including cytokines, chemokines, and immune cells, and thus carry important information regarding the overall health state of the animal. Proteomic profiling of various biological fluids, including blood plasma, has been used as a method for developing biomarkers for a variety of conditions in cattle (Faulkner et al., 2012; Miller et al., 2019). More recently, blood plasma-derived extracellular vesicles (**EV**s) have been the focus of many clinical diagnostic and prognostic studies in relation to their potential as biomarkers of health and disease (Raimondo et al., 2011; Madhavan et al., 2015; Rashed et al., 2017). Small EVs (~50 to 150 nm diameter), termed exosomes (**EX**s), are released by cells into the external milieu and carry molecular cargo such as proteins, nucleic acids, and lipids specific to their cell type of origin (Stoorvogel et al., 2002; Zhang et al., 2019). While they are essential in cell–cell communication and signaling, there is also an increasing body of evidence linking unique EX cargo to cancer and disease in humans (Han et al., 2018; Meng et al., 2018; Riva et al., 2019).

The majority of studies involving EX or other EV subtypes have been performed in relation to human health; however, they have also gained interest in the agricultural sector for their ability to predict or improve fertility in dairy cows (Mitchell et al., 2016; Almughlliq et al., 2018; Wang et al., 2020). To date, no studies have been performed in relation to using blood plasma-derived EXs to identify cattle with inherent susceptibility to tick infestation. As a tool for understanding physiological perturbations leading to susceptibility to disease states, proteomic analysis of EXs is particularly useful, as proteins are the effectors of physiological change within the body. As such, any significant differences in proteomic content between two or more divergent groups may shed light on the underlying mechanisms of, for example, immune dysregulation in cattle with extremely high tick burden as compared with cattle with low or negligible tick burden.

The aim of the present study is to determine whether qualitative proteomic analysis of blood plasma-derived EXs can identify notable differences in cattle with high or low tick burden. The secondary aim of this study is to establish whether proteomic analysis of blood plasma-derived EXs can be used as a tool to assess the physiological effects of high vs. low tick burden in a genetically similar group of beef cattle.

## Material and Methods

### Animals, management, and blood collection

The animals, management, and sample collections were approved by the Animal Welfare Unit, UQ Research and Innovation, the University of Queensland (UQCCR/459/16). A total of 199 animals were selected randomly and tick scores were given as described later. The authors confirm that this study was carried out in compliance with ARRIVE guidelines (https://arriveguidelines.org/arrive-guidelines).

The cattle used in this study were of species *Bos taurus*. All animals were tick exposure naïve, with no previous prophylactic tick treatments. The cattle under study were released into a paddock with known tick prevalence, and natural tick infestation was allowed to occur.

Each cow underwent careful assessment for evidence of tick infestation as part of a thorough physical examination as follows; animals were hand-checked for the presence and absence of ticks on their hind regions and belly over a 3-mo period. A scoring system was developed (1 to 5, A or B): 1) no identifiable tick burden, 2) <10 ticks, 3) 20 to 100 ticks, 4) 100 to 200 ticks, and 5) >200 ticks with (A) representing crusting and (B) no crusting. Animals with a score of <3 were left untreated. Detailed information of cattle histories and other relevant information (e.g., weight and pasture location) was recorded. Cattle from group 1A or B were considered high tick resistant (**HTR**), and those from group 4 or 5A or B were low tick resistant (**LTR**). Three HTR (classification 1B) and three LTR (classification 4B and 5A) cattle (*n* = 6) from the same paddock were chosen at random for this study. The cattle selected were all female, 1.5 ± 0.3 yr of age at the time of baseline tick scoring (no exposure), with weight 362.2 ± 40.6 kg measured at 1.1 ± 0.1 yr of age. Tick scoring was performed every 2 to 3 wk for up to six assessments including baseline score. Cattle with heavy tick burden following first tick exposure were assessed until tick score reached 4A or higher (minimum three separate tick scoring assessments).

Blood was collected from cattle in ethylenediaminetetraacetic acid (**EDTA**) vacutainer tubes. Plasma was separated by centrifugation at 3,000 × *g* for 10 min at 4 °C. The plasma was aspirated and stored at −80 °C until thawed for EV/EX isolation. One 10 mL aliquot of plasma per biological replicate was thawed on ice on the same day as EX isolation and enrichment were initiated.

### EV isolation and enrichment

#### Sequential centrifugation and ultracentrifugation

Ultracentrifugation (**UC**) was performed as previously described (Koh et al., 2018). Briefly, EVs were isolated from 8 mL thawed blood plasma using an established sequential centrifugation protocol. Plasma was centrifuged at 2,000 × *g* for 30 min at 4 °C and 12,000 × *g* for 30 min at 4 °C to remove cellular debris and apoptotic bodies. It was then filtered through a 0.22-μm polyethersulfone membrane filter (Corning Inc., Corning, NY), cleared and filtered blood plasma supernatant was transferred into 32.4-mL OptiSeal Polypropylene Tube (361625, Beckman Coulter), and brought to equal volumes with Dulbecco’s Phosphate Buffered Saline (**DPBS**, pH 7.0 to 7.2) (Vitrolife, Australia). Samples were centrifuged at 100,000 × *g* for 2 h at 4 °C (Beckman, Type 50.2 Ti, Fixed-angle ultracentrifuge rotor). The supernatant was discarded, and the pellet containing EVs was resuspended in 500 µL DPBS. Following UC, samples were stored at −80 °C until the next day.

#### Size exclusion chromatography

Samples were thawed on ice to perform size exclusion chromatography as previously described (Koh et al., 2018). Briefly, the columns and filtered DPBS were brought to room temperature prior to loading the sample onto the column bed. The 500-µL EV sample was loaded onto the column gel bed and 500 µL fractions collected as follows: 1 to 6 as void volume fraction (3 mL total), 7 to 10 as EX fractions, and 11 to 16 as non-EX fractions known to contain soluble plasma proteins, protein aggregates, and nucleic acids. Columns were used up to three times each. In between uses, the columns were flushed with 0.5 mL 0.5M NaOH solution, followed by 15 to 20 mL filtered DPBS. EX fractions 7 to 10 and non-EX fractions 11 to 16 were pooled separately to a final volume 1 mL and used for downstream analyses. The remaining fraction volumes were stored at −80 °C until required.

### Characterization of small EVs (EXs)

#### Protein quantification

The total protein concentrations of pooled EX and non-EX fractions were determined by micro bicinchoninic acid (**BCA** Protein Assay Kit (cat number 23235, Thermofisher Scientific, Australia) following the microplate assay protocol as per the manufacturer’s instructions. Briefly, bovine serum albumin (BSA) standards and EX samples (diluted 1:10) were solubilized 1:1 (v/v) in lysis buffer (1% w/v sodium deoxycholate [**SDC**] and 20 mM Tris-HCl pH 8.5), sonicated in an ice bath for 2 min, and incubated on ice with gentle agitation for 20 min prior to assay. Protein standards were prepared in triplicate and samples in duplicate. About 140 µL of protein standard or sample/lysis buffer was transferred onto a 96 well flat-bottom microplate (N2936, CELLSTAR, Greiner, Sigma) in triplicate (standard) or duplicate (sample). Micro BCA working reagent was added at a ratio of 1:1 with standard/sample and incubated at 37 °C in the dark for 2 h, cooled to room temperature, and absorbance read at 562 nm.

#### Nanoparticle tracking analysis

Nanoparticle tracking analysis (**NTA**) measurements were performed using a NanoSight NS500 instrument (NanoSight NTA 3.1 Build 3.1.46). Instrumentation calibration was performed using 100-nm synthetic beads at a 1:250 dilution. Measurements of samples included particle concentrations and mean and mode sizes of nanoparticles enriched from blood plasma in individual fractions (representative sample) (Supplementary Data File 1, Figure S1).

### Sample preparation for mass spectrometry analysis

#### Filter-aided sample preparation

Pooled 7 to 10 fractions were combined for each method to create a master pool per method and processed for MS analysis using a modified filter-aided sample preparation (Wiśniewski et al., 2009). For the master pool EX samples, a volume of protein extract corresponding to ~10 to 20 µg total protein was mixed at a ratio of 1:1 with lysis buffer (1% w/v SDC, 100 mM dithiothreitol [**DTT**] in 100 mM Tris-HCl pH 8.5, cOmplete-mini EDTA-free protease inhibitor cocktail [Roche]). All samples were sonicated in an ice bath for 2 min and incubated on ice for 20 min. Samples were loaded onto Nanosep Centrifugal Devices with Omega Membrane 30K (PALL) and centrifuged at 14,000 × *g* for 15 min at 21 °C. As EX sample volumes exceeded device capacity, EX samples were loaded onto the device in 450 µL aliquots and centrifuged as described. This was repeated until all the samples had passed through the filter with flow-through discarded. Proteins were reduced by adding 200 μL of DTT–Urea buffer (8M urea, 100 mM Tris-HCl pH 8.5, 25 mM DTT) directly to the filter and incubating for 60 min at room temperature (RT) will gentle agitation. Samples were centrifuged at 14,000 × *g* for 15 min at 21 °C. Filters were washed with 200 μL Urea–Tris buffer (8 M urea, 100 mM Tris-HCl pH 8.5) and centrifuged at 14,000 × *g* for 15 min at 21 °C. Reduced samples were alkylated with 100 μL iodoacetamide (**IAA**)–Urea buffer (50 mM IAA and 8 M Urea–Tris buffer) and incubated at RT for 20 min on agitator. The filters were centrifuged at 14,000 × *g* for 15 min at 21 °C. The filters were washed twice with 200 μL Urea–Tris buffer and centrifuged at 14,000 × *g* for 15 min at 21 °C each. The filters were equilibrated with two washes, 200 μL 100 mM ammonium bicarbonate (**AMBIC**) and centrifugation at 14,000 × *g* for 15 min at 21 °C. Samples were digested overnight (16 h) with trypsin (Trypsin Gold, Mass Spectrometry Grade, Promega) at 37 °C in a humidified chamber with gentle agitation, with a volume of trypsin added at an enzyme to protein ratio of 1:50. The next day, filters were transferred to clean 1.5-mL Eppendorf tubes and peptides collected by centrifugation at 14,000 × *g* for 15 min at 21 °C. One additional elution was performed by adding 60 µL 100 mM AMBIC and centrifugation at 14,000 × *g* for 15 min at 21 °C.

#### Peptide desalting

Peptide digests were acidified by mixing 1:1 with 4% trifluoroacetic acid (**TFA**) solution. StageTips were produced with double strong cation exchange (SCX) membrane (part no: 2251, Empore) as described in Supplementary File 2. About 50 μL of 100% acetonitrile (**ACN**) was passed through the tip using centrifugal force (2 min spin at 300 × *g* rpm) and positive pressure; 50 μL of 5% ammonium hydroxide/80% ACN (Elution buffer) was added to the tips and passed through the tip using centrifugal force and positive pressure; and 50 μL of 0.2% TFA (Wash buffer) was added to the tip and passed through the tip using centrifugal force and positive pressure. Each sample was loaded onto a StageTip and passed through the tip using centrifugal force and positive pressure. About 50 μL of wash buffer was added to the tip and passed through the tip using centrifugal force or positive pressure, three times in total. The tip was placed in a clean 1.5 mL microcentrifuge tube and 80 μl of elution buffer was added to the tip and passed through the tip using centrifugal force or positive pressure. The eluted peptides were dried in a vacuum centrifuge and reconstituted in 15-μL indexed retention time (iRT) buffer (Biognosys-11). 

#### Peptide assay

Samples were assayed for peptide concentration with Pierce Quantitative Colorimetric Peptide Assay according to the manufacturer’s instructions (cat number 23275, Thermofisher Scientific). Peptide concentration in all samples was equalized by an appropriate addition of iRT buffer.

### Mass spectrometry

All peptide samples were analyzed by LC-mass spectrometry (**MS**)/MS as follows. Reversed-phase chromatography was conducted on an Eksigent Ekspert nanoLC 400 System (Eksigent Technologies) using trapping for 3 min at a flow rate of 10 μL/min onto a Trajan ProteCol trap (120 Å, 3 μm, 10 mm × 300 μm) followed by separation on an Eksigent ChromXP C18 3 μm 120 Å (3C18-CL-120, 3 μm, 120 Å, 0.3 × 150 mm) analytical column at a flow rate of 5 μL/min maintained at 40 °C. Trapping utilized mobile phase A only, whereas separation utilized a combination of mobile phases A and B. Mobile phase A consisted of 0.1% FA in water, and mobile phase B was made of 0.1% formic acid (FA) in ACN. Peptides were separated by a 68-min linear gradient of 3% to 25% mobile phase B followed by a 5-min linear gradient of 25% to 35% mobile phase B. After peptide elution, the column was flushed with 80% mobile phase B for 5 min and re-equilibrated with 97% A for 8 min before the next injection. MS was conducted on Triple time-of-flight (**TOF**) 6600 (SCIEX) instrument equipped with DuoSpray Ion Source configured for microflow high performance liquid chromatography (HPLC) applications.

#### Data-dependent acquisition–MS data acquisition

High-resolution (30,000) TOF MS scan was collected over a range of *m/z* 400 to 1,250 for 0.25 s, followed by high-sensitivity TOF MS/MS scans over a range of *m/z* 100 to 1,800 on up to the 30 most abundant peptide ions (0.05 s per each scan) that had an intensity greater than 150 cps and charge state of 2 to 5. The dynamic exclusion duration was set at 15 s. Ion fragmentation in the collision cell used rolling collision energy with the collision energy spread set to 5 eV. The declustering potential was set to 80 V and the remaining gas and source parameters were adjusted as required.

#### Protein identification

MS data files were added to ProteinPilot (v. 5.0.2.0, 5346) and processed individually, using Paragon Algorithm (v. 5.0.2.0, 5174). The fragmentation spectra were searched against cattle proteome (23,847 sequences, downloaded August 2020, available in FASTA format, Uniprot), combined with sequences of cRAP (http://ftp.thegpm.org/fasta/cRAP/) and iRT peptides. The following search parameters were entered: urea denaturation, alkylation with iodoacetamide, species “none,” amino acid substitution, thorough ID, and false discovery rate (**FDR**) analysis 0.01. The protein list was exported to a.xls file, which was subject to an additional refinement. The final list of proteins for each method required a minimum of two peptides per protein ID (1% FDR at the protein level; 5% FDR at the peptide level). All EX fractions were compared with non-EX fractions analyzed in the same way.

### Gene ontology analysis

Proteins identified in ProteinPilot as described above were analyzed for gene ontology (**GO**; PANTHER GO, Gene Ontology Phylogenetic Annotation Project, v 16.0. Available from http://www.pantherdb.org/), including molecular function, cellular component, biological process, pathway analysis, and protein class. Protein accession codes were entered into the search window, searched against species *Bos taurus*, and analyzed for functional classification. FunRich (Functional Enrichment analysis tool) was used to perform enrichment analysis on proteins identified from each enrichment method. The complete Vesiclepedia database (version 4.1, downloaded on July 27, 2021, available from http://microvesicles.org/Archive/VESICLEPEDIA_PROTEIN_MRNA_DETAILS_4.1.txt) was imported into FunRich, and all data were searched against the cattle proteome.

## Results

### Mass spectrometry

A total of 490 unique proteins were detected in EX pooled fractions from HTR and LTR cattle with a 1% FDR cutoff at the protein level (Supplementary Data File 1, Table S1). After an additional FDR cutoff of 5% at the peptide level, 121 proteins were present in all HTR replicates, and 130 proteins in all LTR replicates (Supplementary Data File 1, Figure S2). There were 91 EX proteins shared between HTR and LTR cattle.

### GO analysis

**Figure 1.**
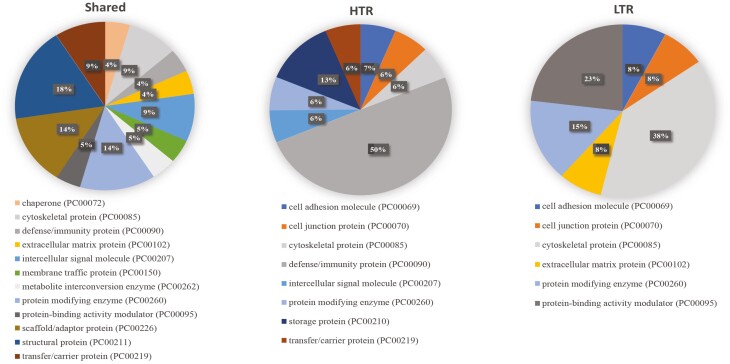
Exosomal proteins by protein class that are shared and unique to high (HTR) and low tick resistant (LTR) cattle. Abbreviation: PC, protein class.

To obtain a clear profile of EX proteins identified in HTR and LTR cattle in association with functions at the molecular, biological, and cellular level, protein UniProt IDs were subjected to GO analysis using an online software tool PANTHER GO (http://www.pantherdb.org/). Shared proteins identified in EX and non-EX fractions were profiled to determine whether there were clear differences in the representation of proteins by protein class (Figure 1A; Supplementary Data File 1, Figure S3). EX shared proteins were divided into 12 separate categories, with structural proteins (18%), scaffold/adapter proteins (14%), and protein-modifying enzymes (14%) accounting for most proteins, with all others being 9% or less. In non-EX fractions, shared proteins were divided into seven different categories, with protein-binding activity modulators (44%), defense/immunity proteins (20%), and transfer/carrier proteins (16%) representing the largest number of proteins, and all others were 8% or less.

EX proteins unique to HTR or LTR cattle were then analyzed in the same way to assess similarities and differences between groups (Figure 1). There were eight protein classes detected in HTR cattle EXs and six in LTR cattle EXs. While 50% of proteins unique to HTR cattle were classified as defense/immunity proteins, this class of protein was not detected in EX proteins unique to LTR cattle. Conversely, protein-binding activity modulator proteins represented 23% of EX proteins unique to LTR cattle, but this protein class was absent in HTR cattle. Cytoskeletal proteins contributed to 38% of unique proteins in LTR cattle, but only 6% unique proteins in HTR cattle. Intercellular signal molecule proteins (6%) and storage proteins (13%) were unique to HTR cattle and absent in LTR cattle.

Next, GO analysis focused on protein families and their subclasses that were associated with EX proteins unique to HTR and LTR cattle (Table 1). HTR cattle EX proteins were associated with immunoglobulins (PC00123) and various immune system processes such as B cell/lymphocyte activation (GO:0042113) and humoral response (GO:0006955), whereas these were absent in LTR cattle. Interestingly, genes associated with vesicle-mediated transport (GO:0006900) were present in HTR cattle and absent from LTR cattle. In LTR cattle, cytoplasmic proteins and their associated processes (GO:0005737) were highly represented, in addition to actin (GO:0051015), carbohydrate derivative (GO:0097367), and genes upstream of adenosine triphosphate (ATP) binding (GO:0017076). 

**Table 1.** Gene ontology (GO) analysis of proteins unique to HTR and LTR cattle

HTR GO analysis		Name	Parent	Child	Families and subfamilies
Category ID	Mapped IDs				
PC00123	BOVIN|Ensembl=ENSBTAG00000045659|UniProtKB=G3N033,BOVIN|Ensembl=ENSBTAG00000050088|UniProtKB=A0A3Q1MI29,BOVIN|Ensembl=ENSBTAG00000050136|UniProtKB=A0A3Q1MSF6,BOVIN|Ensembl=ENSBTAG00000048268|UniProtKB=G3N1H5,BOVIN|Ensembl=ENSBTAG00000003408|UniProtKB=F1MH40,BOVIN|Ensembl=ENSBTAG00000017305|UniProtKB=F1N160,BOVIN|Ensembl=ENSBTAG00000052689|UniProtKB=A0A3Q1MT50	Immunoglobulin	Defense/immunity protein		227
GO:0006955	BOVIN|Ensembl=ENSBTAG00000045659|UniProtKB=G3N033,BOVIN|Ensembl=ENSBTAG00000050136|UniProtKB=A0A3Q1MSF6,BOVIN|Ensembl=ENSBTAG00000003408|UniProtKB=F1MH40,BOVIN|Ensembl=ENSBTAG00000017305|UniProtKB=F1N160,BOVIN|Ensembl=ENSBTAG00000052689|UniProtKB=A0A3Q1MT50	Immune response	Immune system process	Humoral immune response, type-2 immune response, cell activation involved in immune response, innate immune response, adaptive immune response	863
GO:0006900	BOVIN|Ensembl=ENSBTAG00000050088|UniProtKB=A0A3Q1MI29,BOVIN|Ensembl=ENSBTAG00000048268|UniProtKB=G3N1H5	Vesicle budding from membrane	Vesicle-mediated transport	Golgi vesicle budding	813
GO:0005737	BOVIN|Gene=FTH1|UniProtKB=O46414,BOVIN|Ensembl=ENSBTAG00000013343|UniProtKB=O46415,BOVIN|Gene=LIMS1|UniProtKB=F6QGZ0	Cytoplasm	Intracellular	Survival of motor neuron (SMN) complex, endoplasmic reticulum, cell cortex, cytoplasmic vesicle, cytoplasmic microtubule, cytoplasmic region, sarcoplasm, cytosol, cytoplasmic ribonucleoprotein granule, mitochondrion, SMN–Sm protein complex, microbody, endoplasmic reticulum–Golgi intermediate compartment contractile fiber, perinuclear region of cytoplasm, vacuole, membrane coat, Golgi apparatus, phagophore assembly site, soluble N-ethylmaleimide-sensitive factor attachment protein receptor (SNARE) complex, plastid, eukaryotic translation initiation factor 3 complex	24592
GO:0042113	BOVIN|Ensembl=ENSBTAG00000050088|UniProtKB=A0A3Q1MI29,BOVIN|Ensembl=ENSBTAG00000048268|UniProtKB=G3N1H5	B-cell activation	Lymphocyte activation	B-cell proliferation, B-cell activation involved in immune response, B-cell differentiation	150
GO:0006879	BOVIN|Gene=FTH1|UniProtKB=O46414,BOVIN|Ensembl=ENSBTAG00000013343|UniProtKB=O46415	Cellular iron ion homeostasis	Iron ion homeostasis	Iron import into cell	153
LTR GO analysis					
Category ID	Mapped IDs	Name	Parent	Child	Families and subfamilies
GO:0017076	BOVIN|Ensembl=ENSBTAG00000050904|UniProtKB=P62833,BOVIN|Ensembl=ENSBTAG00000006969|UniProtKB=Q2KJD0	Purine nucleotide binding	Nucleotide binding	ATP binding	913
GO:0098797	BOVIN|Ensembl=ENSBTAG00000020645|UniProtKB=A7MBH9,BOVIN|Gene=GNB1|UniProtKB=P62871	Plasma membrane protein complex	Plasma membrane	Plasma membrane respiratory chain complex I, ionotropic glutamate receptor complex, voltage-gated calcium channel complex	594
GO:0005737	BOVIN|Ensembl=ENSBTAG00000017970|UniProtKB=Q08DQ6,BOVIN|Gene=GNB1|UniProtKB=P62871,BOVIN|Ensembl=ENSBTAG00000006969|UniProtKB=Q2KJD0	Cytoplasm	Intracellular	SMN complex, endoplasmic reticulum, cell cortex, cytoplasmic vesicle, cytoplasmic microtubule, cytoplasmic region, sarcoplasm, cytosol, cytoplasmic ribonucleoprotein granule, mitochondrion, SMN-Sm protein complex, microbody, endoplasmic reticulum–Golgi intermediate compartment, contractile fiber, perinuclear region of cytoplasm, vacuole, membrane coat, Golgi apparatus, phagophore assembly site, SNARE complex, plastid, eukaryotic translation initiation factor 3 complex	24592
GO:0097367	BOVIN|Ensembl=ENSBTAG00000050904|UniProtKB=P62833,BOVIN|Ensembl=ENSBTAG00000006969|UniProtKB=Q2KJD0	Carbohydrate derivative binding	Binding	Lipopolysaccharide binding, ATP binding, glycosaminoglycan binding	1109
GO:0051015	BOVIN|Ensembl=ENSBTAG00000008631|UniProtKB=Q92176,BOVIN|Ensembl=ENSBTAG00000021455|UniProtKB=Q5E9F7	Actin filament binding	Actin binding		473

Abbreviations: HTR, high tick resistance; LTR, low tick resistance; PC, protein class.

Finally, we examined the pathways associated with EX proteins unique to HTR and LTR cattle to assess whether these may have an association with tick burden (Supplementary Data File 1, Tables S2 and S3). While there were only 8 pathways associated with proteins unique to HTR cattle, there were 45 pathways associated with proteins unique to LTR cattle. Pathways involving three or more genes were considered enriched. Of these, pathways related to inflammation (P00031), G-protein signaling (P00026), and cytoskeletal regulation (P00016) were enriched in LTR cattle, whereas only the integrin signaling pathway (P00034) was enriched in HTR cattle EXs.

### Functional enrichment analysis

Differences in EX protein composition between HTR and LTR cattle may translate to functional differences associated with tick burden. To explore differences in protein composition as related to function, the FunRich software analysis tool (http://funrich.org/index.html) was used to perform functional enrichment analysis of EX proteins in HTR and LTR cattle.

Several differences were identified between HTR and LTR cattle in various categories of protein function (Supplementary Data File 1, Figures S4 and S5). Biological processes relating to iron ion transport and immune response contributed to a significantly larger percentage of proteins in HTR cattle compared with LTR cattle (4.82% vs. 1.19% and 10.84% vs. 2.38%, respectively). The calcium-binding epidermal growth factor (**EGF–CA**) protein domain was increased nearly 2-fold in proteins identified in LTR cattle (7.41% vs. 3.80%), whereas immunoglobulin (**IG**) and IG variable (v)-type domains were increased 3-fold in proteins identified in HTR cattle (11.39% vs. 3.70% in both cases). The IG-constant 1 (c1)-type domain was also increased in HTR cattle (11.39% vs. 8.64%).

## Discussion

Plasma-derived EXs are diverse in origin and can provide essential information regarding the health status of the animal. This is the first study that has utilized MS to analyze the proteomic cargo of EXs in relation to tick burden in cattle. Regarding the first aim of the present study, we have provided evidence that the MS analysis of the EX–proteome derived from HTR and LTR cattle plasma identified several differences that indeed correlate with tick burden status. The secondary aim of this study addressed the validity of EX analysis as a novel screening tool for assessing the physiological effects of tick burden. Analysis of EX proteomic cargo identified protein classes, pathways, and protein domains that are directly related to immune status and function. Therefore, EX proteomic cargo analysis provides a previously unused method for studying and understanding biological perturbations associated with tick infestation.

### Increased EGF–CA protein domains may result in widespread alterations to signaling pathways

Protein domains are substructures of proteins that allow for a diverse array of functions within the same molecule. They may give clues to alterations in protein function resulting from evolutionary changes that occur via the incorporation of additional domains at the genetic level or represent unique posttranslational modifications of existing domains. In this study, the number of proteins containing the protein domain EGF-CA was 2-fold increased in LTR cattle. The EGF–CA protein domain is a recent evolutionary adaptation (Wouters et al., 2005). It has been studied with regard to its effects on blood coagulation, and genetic mutations produce biologically inactive proteins that give rise to developmental and clotting disorders in humans (Stenflo et al., 2000). In cattle, the expression of genes associated with Ca^2+^ signaling was found to be increased in the skin of HTR cattle (Bagnall et al., 2009). As ion channels can import Ca^2+^ directly into cells, there may be direct or indirect stimulation of Ca^2+^ signaling in HTR cattle. Additionally, as Ca^2+^-binding EGF domains represent >25% of the 600+ identified EGF modules, any small change in EGF–CA domains between groups may have a significant impact on the multitude of biological pathways in which they participate (Stenflo et al., 2000). If the observed increase in EGF–CA protein domains in LTR cattle is indeed a result of genetic variance, this theory is somewhat supported by the apparent innate tick resistance difference between cattle breeds, suggestive of a link to genetic traits in determining natural resistance (Jonsson et al., 2014). While this is not a definitive finding, further investigation of the EGF–CA domain in follow-on proteomic or genomic studies would be beneficial to better understand the physiological effects of alterations in this domain prevalence and its relationship to tick resistance.

### EX proteome profiling identifies a significant reduction of proteins associated with defense/immunity in LTR cattle

A predictable consequence of tick infestation in cattle is the immunosuppressive effects of heavy tick burden on the animal (Inokuma et al., 1993). EX proteins unique to LTR cattle did not fall into the defense/immunity protein class, which signifies that there is indeed a physiological shift associated with a high tick burden. The reduction of IG, IGv, and IGc1 protein domains in LTR cattle is in further support of impaired immune function in LTR cattle. Of note, EXs from HTR cattle were found to contain proteins related to B-cell and T-cell activation, whereas this was not present in the LTR group. Whether this immune dysregulation is innate or acquired through heavy tick burden is unclear; however, further studies may wish to analyze plasma EXs of cattle prior to tick infestation at a baseline stage, as this will provide an unbiased assessment of natural tick resistance in cattle. This study has demonstrated the ability of EX to carry vital immune proteins, in addition to validating the use of EX as a tool for the physiological assessment of HTR and LTR cattle.

It is known that immune cells such as B and T cells are capable of producing EV, including EX (Zhou et al., 2020). What is currently unknown is the proportion of EXs in blood plasma that are produced by immune cells vs. those that are produced by other cell types and released into systemic circulation. Interestingly, EV can mediate both innate immune activation and immunosuppression (Zhou et al., 2020). EVs interact with antigen-presenting cells via a range of uptake mechanisms to produce an immune response (Robbins and Morelli, 2014; Zhou et al., 2020). As such, they are of ongoing interest in studies related to immune function. The suppression of immune response in the LTR group correlates with heavy tick burden, and potentially a reduction of immune cell-derived EVs in the blood plasma of LTR cattle. This idea is further supported by the significant loss of immune proteins identified in EXs from LTR cattle. The divergent EX immune profiles of HTR and LTR cattle in this study suggest that circulating EXs play an important part in mediating the immune response to tick exposure and follow-on studies may delineate their role in this regard.

## Conclusion

For the first time, we have successfully applied an MS workflow to analyze the proteomic cargo in the blood plasma EXs of cattle with a high or low tick burden. The divergent EX proteomic profiles established using this method are proof of principle for establishing a blood plasma-derived EX screening tool for cattle with a predisposition to tick infestation, which may be of great benefit to cattle farmers when implementing tick management strategies. Sampling cattle at regular timepoints from birth through to adulthood and past first tick exposure would further clarify any biological, physiological, or pathological processes that are either genetically or environmentally acquired leading to increased tick resistance or susceptibility. As this is a global issue, there would be significant financial and environmental gains associated with improvements in diagnostics or prognostics for tick resistance. Future studies may focus on quantitative proteomics analyses of blood plasma-derived EXs to identify differences in shared proteins between HTR and LTR cattle. Quantitative differences in shared proteins can be developed into biomarker panels and further assist in determining the resistance status of cattle at earlier timepoints and thus warrant further investigation.

## Supplementary Material

skac015_suppl_Supplementary_Material

skac015_suppl_Supplementary_Data

skac015_suppl_Supplementary_File_2

## Abbreviations

ACN: acetonitrile
AMBIC: ammonium bicarbonate
BCA: bicinchoninc acid
BSA: bovine serum albumin
DPBS: Dulbecco’s phosphate buffered saline
DTT: dithiothreitol
EDTA: ethylenediaminetetraacetic acid
EGF: epidermal growth factor
EGF–CA: epidermal growth factor-calcium binding
EV: extracellular vesicle(s)
EX: exosome(s)
FDR: false discovery rate
GO: gene ontology
HTR: high tick resistance
IAA: iodoacetamide
IG: immunoglobulin
IGc1: immunoglobulin constant type-1
IGv: immunogloublin variable type
LTR: low tick resistance
MS: mass spectrometry
NTA: nanoparticle tracking analysis
SDC: sodium deoxycholate
TFA: trifluoroacetic acid;
TOF: time-of-flight
UC: ultracentrifugation
