# Supplementary material for: Proteomic profiling of plasma-derived small extracellular vesicles: a novel tool for understanding the systemic effects of tick burden in cattle
Source: J Anim Sci. 2022 Jan 19;100(2):skac015. doi: 10.1093/jas/skac015 (PMC8867580; doi:10.1093/jas/skac015)
Supplement: skac015_suppl_Supplementary_Material [file skac015_suppl_Supplementary_Material.docx]

# Supplementary data

**Table S1:** Number of protein identifications in exosomal pooled fractions 7 – 10 in HTR and LTR cattle.

| **List names** | **number of elements** | **number of unique elements** |
| --- | --- | --- |
| Low tick 1 | 283 | 89 |
| Low tick 2 | 269 | 73 |
| Low tick 3 | 200 | 28 |
| High tick 1 | 236 | 58 |
| High tick 2 | 194 | 38 |
| High tick 3 | 236 | 49 |
| **Overall number of unique elements** |  | **490** |

**Figure S1:** Representative nanoparticle tracking analysis of exosomal fractions 6 – 16 resulting from size exclusion chromatography. F = Fraction.

**
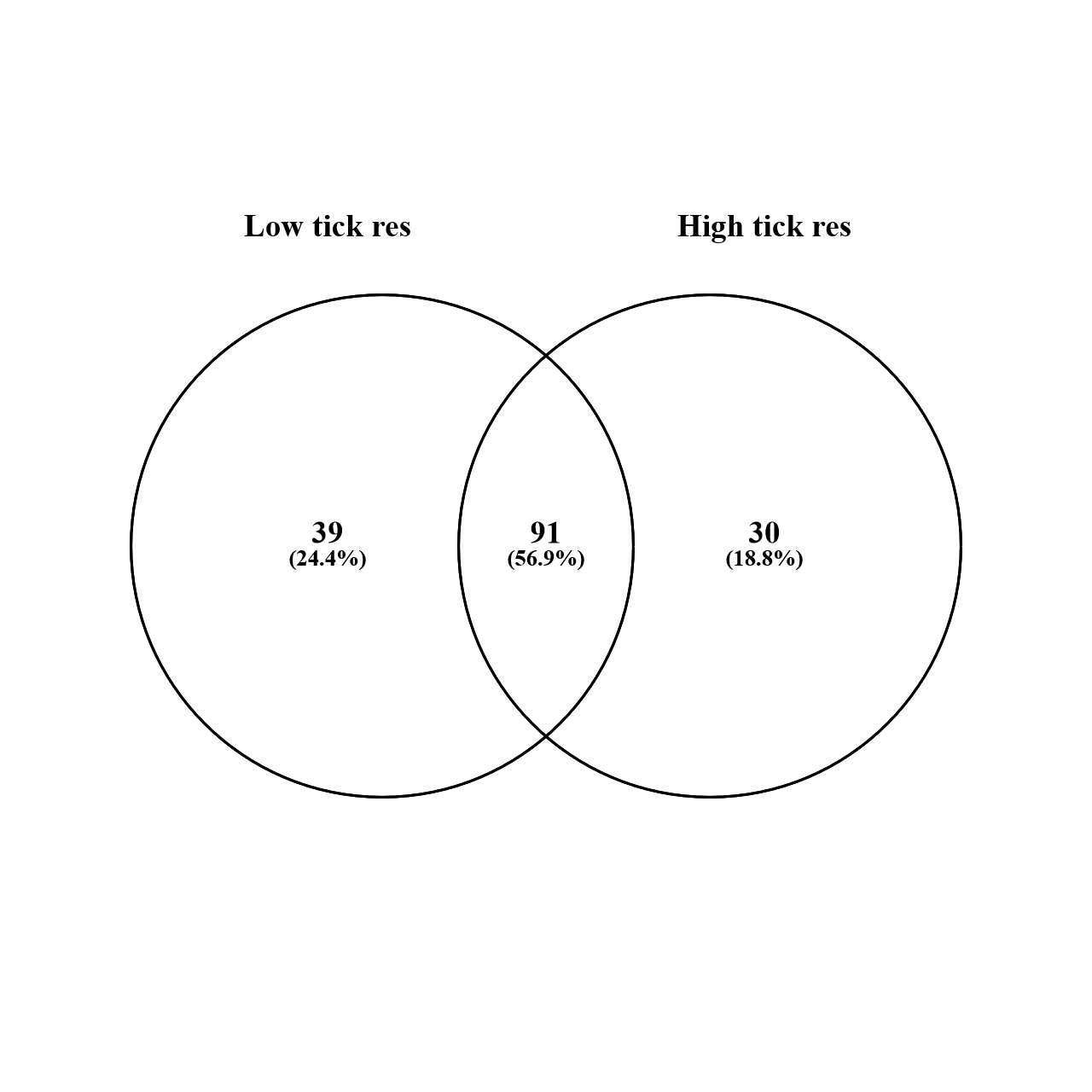
Figure S2:** Venn diagram of shared and unique proteins identified in exosomal pooled fractions 7 – 10 in high (right) and low (left) tick resistant cattle.


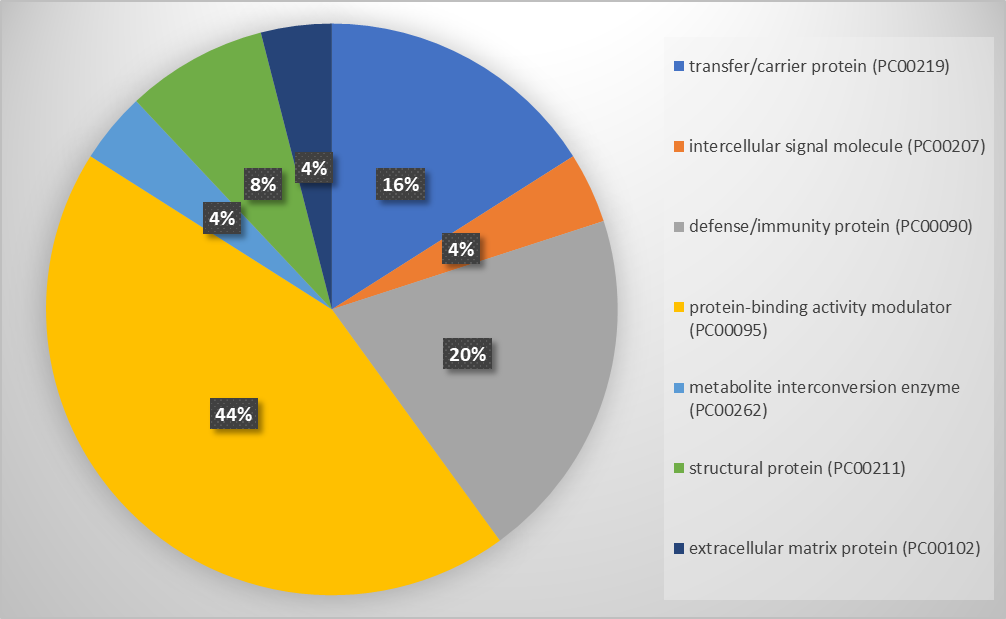
**Figure S3:** Non-exosomal shared proteins identified in pooled fractions 11 – 16 by protein class (PC).

**Table S2: HTR signalling pathways. Green indicates pathways involving 3 or more genes and are considered enriched.**

| **Pathway** | **Genes involved** | **% of total genes** | **% of total pathways** |
| --- | --- | --- | --- |
| Alzheimer disease-presenilin pathway (P00004) | 1 | 5.60% | 8.30% |
| Blood coagulation (P00011) | 1 | 5.60% | 8.30% |
| Cadherin signaling pathway (P00012) | 1 | 5.60% | 8.30% |
| Cytoskeletal regulation by Rho GTPase (P00016) | 1 | 5.60% | 8.30% |
| Huntington disease (P00029) | 1 | 5.60% | 8.30% |
| Inflammation mediated by chemokine and cytokine signaling pathway (P00031) | 1 | 5.60% | 8.30% |
| Integrin signalling pathway (P00034) | 4 | 22.20% | 33.30% |
| Nicotinic acetylcholine receptor signaling pathway (P00044) | 1 | 5.60% | 8.30% |
| Wnt signaling pathway (P00057) | 1 | 5.60% | 8.30% |

**Table S3: LTR signalling pathways.**

| **Pathway** | **Genes involved** | **% total genes** | **% total pathways** |
| --- | --- | --- | --- |
| 5HT1 type receptor mediated signaling pathway (P04373) | 2 | 9.10% | 3.00% |
| 5HT2 type receptor mediated signaling pathway (P04374) | 1 | 4.50% | 1.50% |
| 5HT4 type receptor mediated signaling pathway (P04376) | 1 | 4.50% | 1.50% |
| Alzheimer disease-presenilin pathway (P00004) | 1 | 4.50% | 1.50% |
| Angiogenesis (P00005) | 2 | 9.10% | 3.00% |
| Angiotensin II-stimulated signaling through G proteins and beta-arrestin (P05911) | 1 | 4.50% | 1.50% |
| B cell activation (P00010) | 1 | 4.50% | 1.50% |
| Beta1 adrenergic receptor signaling pathway (P04377) | 1 | 4.50% | 1.50% |
| Beta2 adrenergic receptor signaling pathway (P04378) | 1 | 4.50% | 1.50% |
| Beta3 adrenergic receptor signaling pathway (P04379) | 1 | 4.50% | 1.50% |
| Cadherin signaling pathway (P00012) | 1 | 4.50% | 1.50% |
| CCKR signaling map (P06959) | 2 | 9.10% | 3.00% |
| Cortocotropin releasing factor receptor signaling pathway (P04380) | 1 | 4.50% | 1.50% |
| Cytoskeletal regulation by Rho GTPase (P00016) | 3 | 13.60% | 4.50% |
| Dopamine receptor mediated signaling pathway (P05912) | 2 | 9.10% | 3.00% |
| Endogenous cannabinoid signaling (P05730) | 1 | 4.50% | 1.50% |
| Enkephalin release (P05913) | 2 | 9.10% | 3.00% |
| GABA-B receptor II signaling (P05731) | 1 | 4.50% | 1.50% |
| Gonadotropin-releasing hormone receptor pathway (P06664) | 2 | 9.10% | 3.00% |
| Heterotrimeric G-protein signaling pathway-Gi alpha and Gs alpha mediated pathway (P00026) | 3 | 13.60% | 4.50% |
| Heterotrimeric G-protein signaling pathway-Gq alpha and Go alpha mediated pathway (P00027) | 2 | 9.10% | 3.00% |
| Heterotrimeric G-protein signaling pathway-rod outer segment phototransduction (P00028) | 1 | 4.50% | 1.50% |
| Histamine H1 receptor mediated signaling pathway (P04385) | 1 | 4.50% | 1.50% |
| Histamine H2 receptor mediated signaling pathway (P04386) | 1 | 4.50% | 1.50% |
| Huntington disease (P00029) | 1 | 4.50% | 1.50% |
| Inflammation mediated by chemokine and cytokine signaling pathway (P00031) | 3 | 13.60% | 4.50% |
| Integrin signalling pathway (P00034) | 2 | 9.10% | 3.00% |
| JAK/STAT signaling pathway (P00038) | 1 | 4.50% | 1.50% |
| Metabotropic glutamate receptor group II pathway (P00040) | 2 | 9.10% | 3.00% |
| Metabotropic glutamate receptor group III pathway (P00039) | 2 | 9.10% | 3.00% |
| Muscarinic acetylcholine receptor 1 and 3 signaling pathway (P00042) | 1 | 4.50% | 1.50% |
| Muscarinic acetylcholine receptor 2 and 4 signaling pathway (P00043) | 2 | 9.10% | 3.00% |
| Nicotine pharmacodynamics pathway (P06587) | 1 | 4.50% | 1.50% |
| Nicotinic acetylcholine receptor signaling pathway (P00044) | 1 | 4.50% | 1.50% |
| Notch signaling pathway (P00045) | 1 | 4.50% | 1.50% |
| Opioid prodynorphin pathway (P05916) | 2 | 9.10% | 3.00% |
| Opioid proenkephalin pathway (P05915) | 2 | 9.10% | 3.00% |
| Opioid proopiomelanocortin pathway (P05917) | 2 | 9.10% | 3.00% |
| Oxytocin receptor mediated signaling pathway (P04391) | 1 | 4.50% | 1.50% |
| p38 MAPK pathway (P05918) | 1 | 4.50% | 1.50% |
| PI3 kinase pathway (P00048) | 2 | 9.10% | 3.00% |
| T cell activation (P00053) | 1 | 4.50% | 1.50% |
| Thyrotropin-releasing hormone receptor signaling pathway (P04394) | 1 | 4.50% | 1.50% |
| VEGF signaling pathway (P00056) | 1 | 4.50% | 1.50% |
| Wnt signaling pathway (P00057) | 2 | 9.10% | 3.00% |

**
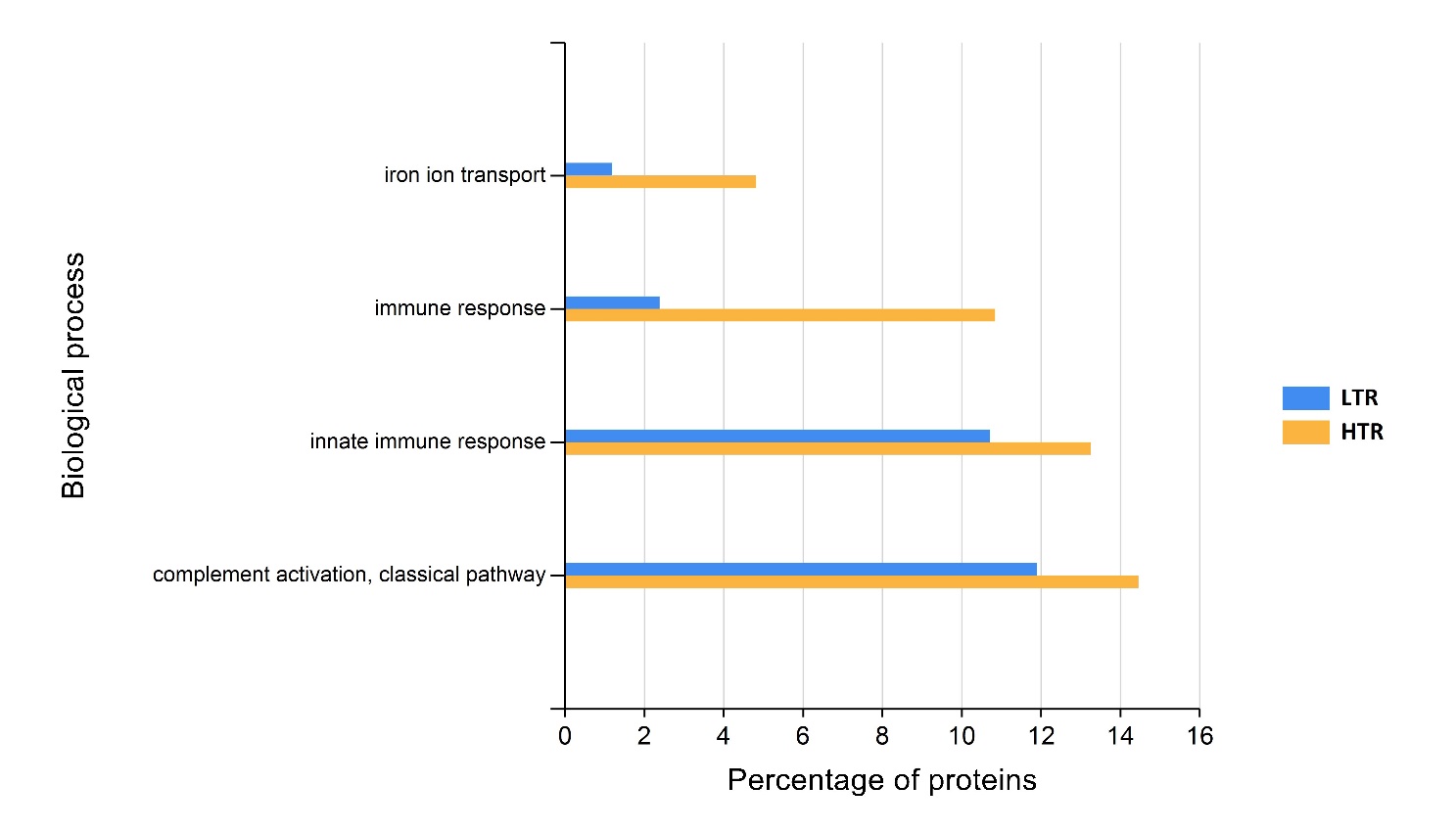
Figure S4:** FunRich analysis of Biological Process in HTR and LTR cattle.

**
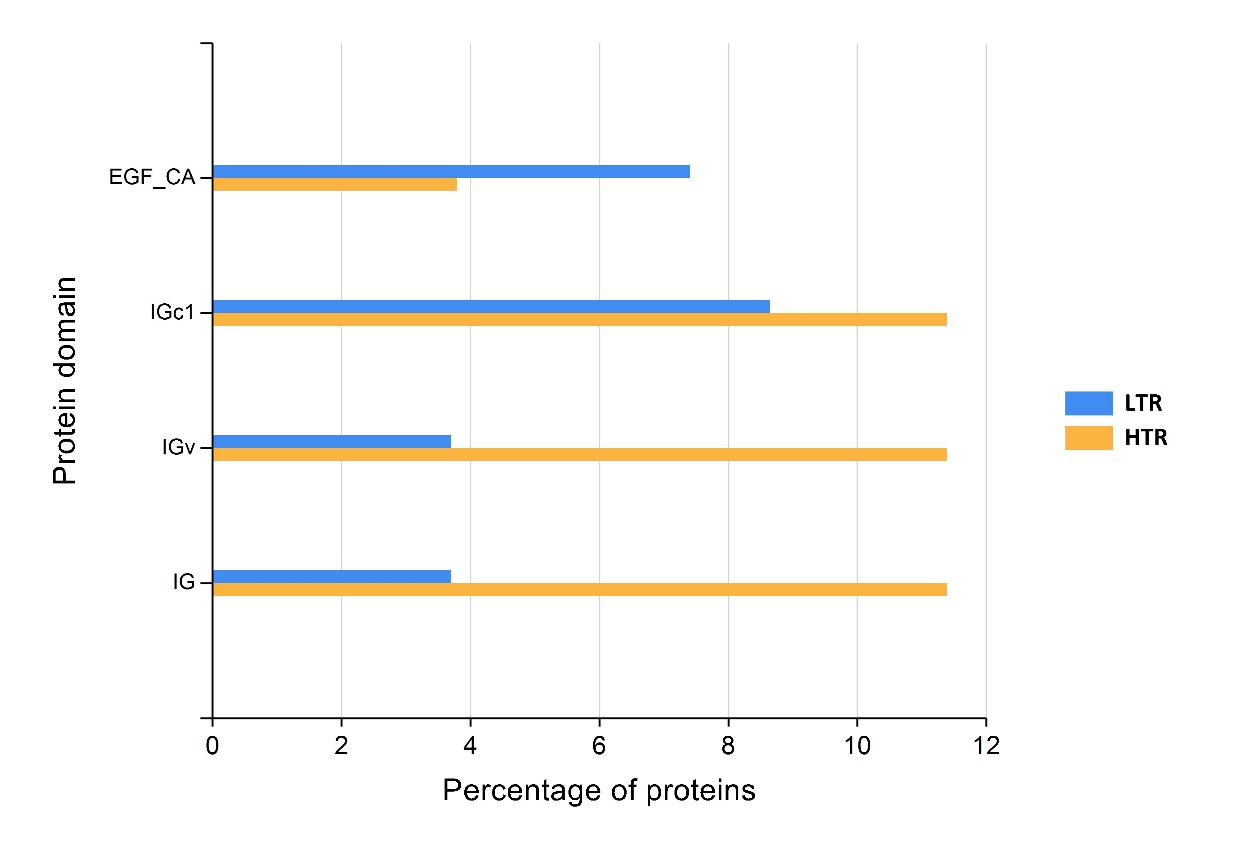
**

**Figure S5:** FunRich analysis of Protein Domain in HTR and LTR cattle.
