# Supplementary material for: Proteomic profiling of plasma-derived small extracellular vesicles: a novel tool for understanding the systemic effects of tick burden in cattle
Source: J Anim Sci. 2022 Jan 19;100(2):skac015. doi: 10.1093/jas/skac015 (PMC8867580; doi:10.1093/jas/skac015)
Supplement: skac015_suppl_Supplementary_File_2 [file skac015_suppl_Supplementary_File_2.docx]

## Production of StageTips.

Two Empore SCX membrane disks were placed on top of each other and on top of a paper disk in a clean Petri dish. A blunt-end 18g needle was attached to a 1 mL syringe and filled with air. The needle was pressed firmly into the membrane and rotated clockwise to cut out the disks. The syringe and needle containing the double SCX membrane material was inserted into a 300 uL white robotic tip (Eppendorf, cat no: 0030 014.464) and the plunger depressed rapidly down to create positive pressure and eject the membrane into the pipette tip. This was repeated, if necessary, until the double membrane was sitting firmly at the end of the pipette tip.
